# Supplementary material for: Patterns of Intron Gain and Loss in Fungi
Source: PLoS Biol. 2004 Nov 30;2(12):e422. doi: 10.1371/journal.pbio.0020422 (PMC532390; doi:10.1371/journal.pbio.0020422)
Supplement: Table S1 — Also available at http://genes.mit.edu/NielsenEtAl/. (4.3 MB ZIP). [file pbio.0020422.st001.zip › NielsenEtAl/html/1281.html]

AN6083.1.NCU08964.1.MG03136.1.FG10246.1


```
 CLUSTAL W (1.82) Multiple Sequence Alignments - Introns Inserted


Sequence 1: NCU08964.1	221 aa
Sequence 2: MG03136.1	219 aa
Sequence 3: FG10246.1	221 aa
Sequence 4: AN6083.1	250 aa
Alignment Length: 250 aa
Number Identitical Residues: 173 aa
Alignment Score (without introns) 7540


MG03136.1 	MARRPARCYRYCKNK0PYPKSRFNRGVPDPKIRIFDLGRKRATVDDFPLCIHLVSNEYEQ
NCU08964.1	MARRPARCYRYCKNK0PYPKSRFNRGVPDPKIRIFDLGRKRATVDDFPLCIHMVSNEYEQ
FG10246.1 	MARRPARCYRYCKNK0PYPKSRFNRGVPDPKIRIFDLGRKRANVDDFPLCIHLVSNEYEQ
AN6083.1  	MARRPARCYRYCKNK0PYPKSRFNRGVPDPKIRIFDLGRKKANVDDFPLCVHLVSNEYEQ
          	*************** ************************:*.*******:*:*******

MG03136.1 	LSSEALEAARICAN-----------------------------K2YLVKNAGKEGFHLRV
NCU08964.1	LSSEALEAARICAN-----------------------------K~YLVKTAGKEGFHLRV
FG10246.1 	LSSEALEAARICAN-----------------------------K~YLVKNTGKEGFHLRV
AN6083.1  	LSSEALEAARICANKYVLTATEPDFRDEKKDMRREETILTMDYR~YLVKIAGKEGFHLRV
          	**************.   :::...  .....   ..:  : . : **** :*********

MG03136.1 	RAHPYHVVRINKMLSCAGADRLQTGMRGAWGKPNGTVARVNIGQIILSVRTRDSN1RALA
NCU08964.1	RVHPYHVVRINKMLSCAGADRLQTGMRGAWGKPNGTVARVNIGQIIMSVRTRDSN1KAFA
FG10246.1 	RAHPFHVVRINKMLSCAGADRLQTGMRGAWGKPNGTVARVNIGQILMSVRTRDAN1RAIA
AN6083.1  	RVHPFHVIRINKMLSCAGADRLQTGMRGAFGKPQGKVARVNIGQIILSVRTRDSH~RATA
          	*.**:**:*********************:***:*.*********::******:: :* *

MG03136.1 	LEALRRSQYKFPGRQKIIISKNWGFTPLRREEYLEKKAAGRVKVDGAYVQFLANHGSLEN
NCU08964.1	LEALRRSQYKFPGRQKIIISKNWGFTPLRREEYLEKKAAGKLLIDGAYVQFLGNRGSLER
FG10246.1 	LEALRRSQYKFPGRQKIIVSKNWGFTPLRREEYLDKKAAGRVKVDGAYVQFLSNHGSLER
AN6083.1  	IEALRRSMYKFPGRQKIIVSKNWGFTPVRREDYVQLRQEGKLKQDGAYVQFLRGHGQIEE
          	:****** **********:********:***:*:: :  *::  ******** .:*.:*.

MG03136.1 	NMKRFPEAFAA--
NCU08964.1	NMKRFPDAFATEA
FG10246.1 	NIRRFPDAFKSEA
AN6083.1  	NMRRFPDAYSTEA
          	*::***:*: :.:
```
